# Memory regulatory T cells in pregnancy

**DOI:** 10.3389/fimmu.2023.1209706

**Published:** 2023-10-26

**Authors:** Zeyang Chen, Yanan Zhang, Joanne Kwak-Kim, Wenjuan Wang

**Affiliations:** ^1^ School of Medicine, Qingdao University, Qingdao, China; ^2^ Reproduction Medical Center, Xinhua Hospital Affiliated to Shanghai Jiaotong University School of Medicine, Shanghai, China; ^3^ School of Integrated Chinese and Western Medicine, Hunan University of Chinese Medicine, Changsha, China; ^4^ Reproductive Medicine and Immunology, Obstetrics and Gynecology, Clinical Sciences Department, Chicago Medical School, Rosalind Franklin University of Medicine and Science, Vernon Hills, IL, United States; ^5^ Center for Cancer Cell Biology, Immunology and Infection, Chicago Medical School, Rosalind Franklin University of Medicine and Science, North Chicago, IL, United States

**Keywords:** memory regulatory T cells, reproductive immunology, pregnancy, recurrent pregnancy loss, gestational diabetes mellitus, preeclampsia

## Abstract

Pregnancy requires the process of maternal immune tolerance to semi-allogeneic embryos. In contrast, an overreactive maternal immune system to embryo-specific antigens is likely to result in the rejection of embryos while damaging the invading placenta, such that the likelihood of adverse pregnancy outcomes can be increased. Regulatory T cells (Tregs) are capable of suppressing excessive immune responses and regulating immune homeostasis. When stimulating Tregs, specific antigens will differentiate into memory Tregs with long-term survival and rapid and powerful immune regulatory ability. Immunomodulatory effects mediated by memory Tregs at the maternal-fetal interface take on critical significance in a successful pregnancy. The impaired function of memory Tregs shows a correlation with various pregnancy complications (e.g., preeclampsia, gestational diabetes mellitus, and recurrent pregnancy losses). However, the differentiation process and characteristics of memory Tregs, especially their role in pregnancy, remain unclear. In this study, a review is presented in terms of memory Tregs differentiation and activation, the characteristics of memory Tregs and their role in pregnancy, and the correlation between memory Tregs and pregnancy complications. Furthermore, several potential therapeutic methods are investigated to restore the function of memory Tregs in accordance with immunopathologies arising from memory Tregs abnormalities and provide novel targets for diagnosing and treating pregnancy-associated diseases.

## 1 Introduction

“Immunological memory” refers to an immune response occurs after the first contact with a specific antigen during the immunization process. When stimulated by the identical antigen again, immunological memory is capable of quickly initiating secondary immunity and inducing a stronger immune response (1). Memory lymphocytes comprise memory B, T, and natural killer (NK) cells which induce stable transcriptional, epigenetic, and metabolic changes besides the rapid expansion of antigen-specific cells (2–4). Notably, T regulatory cells (Tregs) are a specialized subset of CD4^+^T cells characterized by the expression of the X-chromosome-encoded lineage-specific transcription factor, forkhead box protein p3 (Foxp3) (5).

Sir Peter Medawar described a fetus as an allograft developing in an immunocompetent maternal host (6). Before conception, the maternal immune system is exposed to paternal antigens in the semen (7). During embryo implantation, paternal antigens carried by trophoblasts of the placenta come into direct contact with the immune cells at the maternal-fetal interface. It is noteworthy that paternal antigens remain in the maternal peripheral circulation, which has been reported in subsequent pregnancy and even several years after parturition (8, 9). An overreaction of the maternal immune system to paternal (embryo-specific) antigens may result in rejection and damage to the embryo and adverse pregnancy outcomes. Regulatory T cells (Tregs) are capable of mediating immunomodulatory effects at the maternal-fetal interface, which take on critical significance during embryo implantation and subsequent pregnancy maintenance (10). Down-regulated paternal antigen-specific Tregs in peripheral blood show a correlation with multiple pregnancy complications, comprising preeclampsia (PE), preterm birth, as well as spontaneous abortion (11–16). Paternal antigen-specific Tregs persisted at high levels after delivery in mice models and continued to exert tolerance to paternal antigens. When the mice are pregnant again, Tregs are proliferated rapidly and exert stronger maternal-fetal immune tolerance under the effect of paternal antigen-specific Foxp3^+^T cells retained from the previous pregnancy, suggesting that Foxp3^+^T cells during pregnancy exhibit a memory immunoregulatory function (17).

The relevance of memory Tregs to pregnancy outcomes and complications has aroused wide attention and explored over the past few years. Our previous study revealed that the frequency and regulatory capacity of memory Tregs in the peripheral blood of recurrent pregnancy loss (RPL) patients were compromised, suggesting that paternal antigen-specific memory Tregs play an important role in sustaining the pregnancy. Consequently, the disruption of memory Tregs may be implicated in the pathogenesis of RPL (18). Following the mouse and human research, this review illustrates mTreg subtypes, their role in immune tolerance in pregnancy and the dysregulated function, and several mTreg subtypes present in a wide variety of pregnancy complications. Furthermore, controversies about memory Tregs and possible therapeutic methods targeting memory Tregs are discussed.

## 2 Immune memory function of Tregs

### 2.1 Memory subsets of Tregs

Tregs fall into thymus-derived Foxp3^+^ Tregs (tTregs) differentiated from thymic precursor T cells and peripheral naïve CD4^+^ T cell-derived Foxp3^+^ Tregs (pTregs) in accordance with the source (19–21). Naive T cells stimulated by TGF-β and IL-2 are capable of producing Foxp3^+^ Tregs *in vitro* that are termed induced Tregs (22). However, Treg subtypes are not limited to their sources but to their surface markers and functions (e.g., T helper-like Tregs, CD8^+^ Tregs, and Tregs with memory phenotypes) (23–25). It is noteworthy that memory Tregs fall into CD45RA^+^ naive Tregs and CD45RO^+^ memory Tregs following the expression of the cell memory surface marker CD45RO in humans (26). CD45RO^+^ memory Tregs are considered a subgroup of Tregs with activation and functional differentiation since they outperform naive Tregs in the immune regulation and migration ability for local immunotropism (27).

### 2.2 The differentiation regulation of memory Tregs

**Figure 1:**
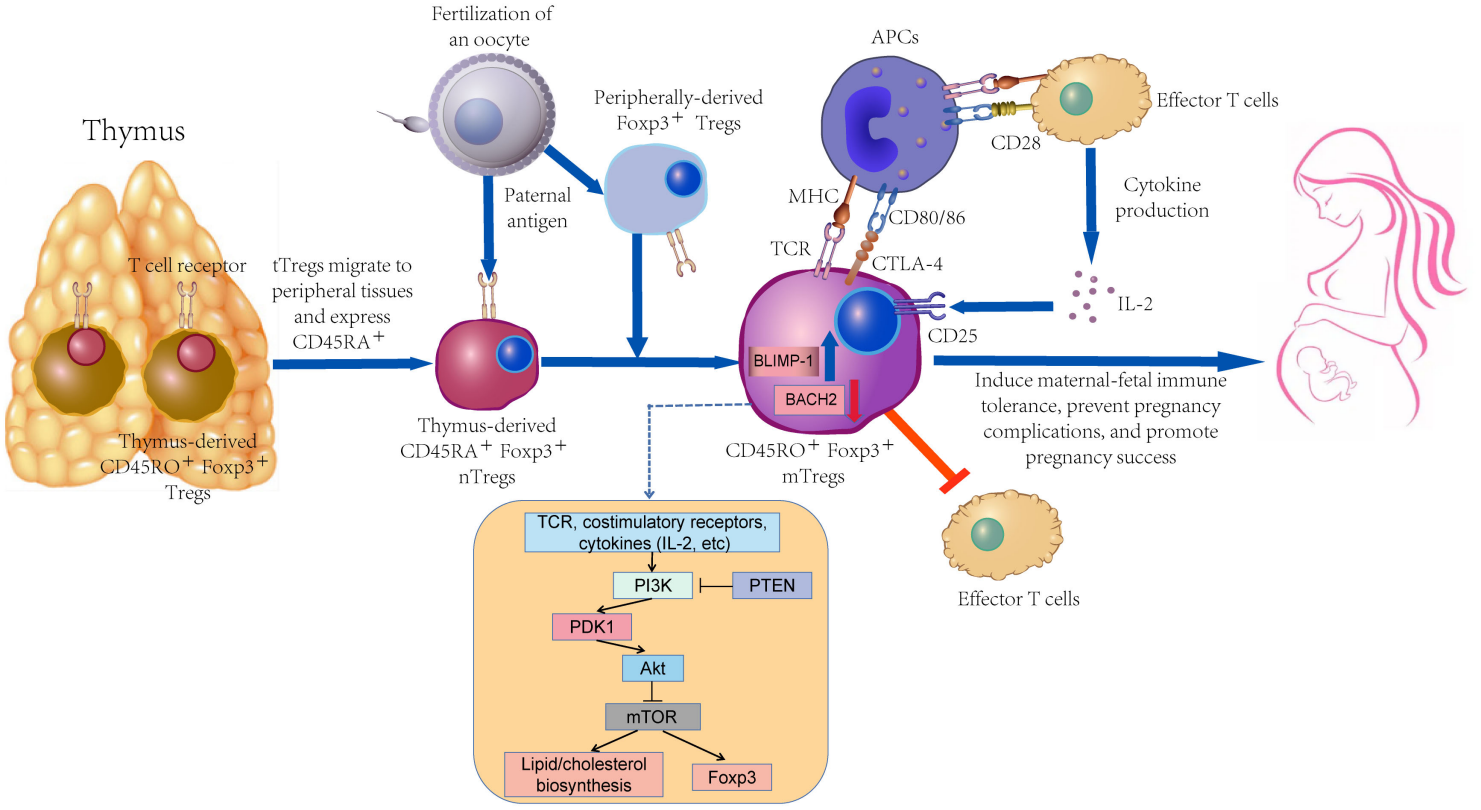
The differentiation process of memory Tregs and their function in pregnancy.

As depicted in **Figure 1**, memory Tregs primarily originate from naive Tregs. They should be further stimulated by continuous TCR signal for exerting a high-effectiveness immunosuppressive function *in vitro* and expressing activation markers (e.g., CD25, GITR, CD95, ICOS, CTLA-4, and Ki67) (28–30). In a physiological polyclonal environment, the activation status of individual Tregs may be affected by the affinity and availability of TCRs to their cognate antigens and the strength and duration of TCR signaling (31). Nevertheless, TCR signaling strength does not exert any effect on the resting/activated Tregs ratio, whereas it controls the activation of Tregs (32).

Differentiation of mTreg is regulated by the transcription factors BACH2 and BLIMP-1 (encoded by PRDM1) (31). The above-mentioned two key transcription factors display a clear, mutually exclusive expression pattern. BACH2 mRNA is highly expressed in naive cells and gradually declines during the differentiation to a memory phenotype, accompanied by a gradual increase in PRDM1 expression. The dynamic interplay between BACH2 and BLIMP-1 was more pronounced in *in-vitro* validation experiments, supporting their role as master regulators of transcriptional programs associated with activation of human Treg differentiation in response to antigenic stimulation (33).

The differentiation of memory Tregs may depend on mTOR signal, and such a process may cover a wide variety of factors (e.g., metabolic processes). With the development of CD8^+^ T cells, effector cell differentiation is more significantly dependent on aerobic glycolysis, whereas memory cell differentiation relies on fatty acid oxidation (34). However, the energy requirement for Tregs is not dependent on glucose transporter-1, whereas it preferentially relies on high lipid oxidation levels. mTOR serves as a critical regulator of T cell metabolism that can integrate nutrient sensing pathways and signaling pathways involved in T cell differentiation, growth, survival, and proliferation (35). TCR, costimulatory molecules, and various cytokines tune the mTOR signal via the upstream PI3K/Akt pathway to content the energy demands associated with T cell activation (36). Moreover, autophagy is active in Tregs while supporting their lineage stability and survival fitness. Tregs-specific deletion of the basic autophagy genes ATG7 or ATG5 will trigger the loss of Tregs. Deficiency in autophagy can up-regulate the metabolic regulators mTORC1 and c-Myc, as well as glycolysis, such that Tregs function is impaired (37). Autophagy in extravillous trophoblast (EVT) cells is reduced in PE patients compared with healthy pregnant women. The invasion and vascular remodeling of autophagy-deficient EVT cells are notably reduced under hypoxic conditions, which may play a certain role in the pathogenesis of PE (38). Previous research has suggested that the inhibition of mTOR during T cell activation can facilitate the production of long-lived Tregs with memory-like phenotype in mice while up-regulating Foxp3 expression (39, 40).

Aryl hydrocarbon receptor (AHR) inhibitor is up-regulated in CD45^+^RA^-^ Tregs, suggesting that Tregs may be differentiated into memory phenotype by regulating AHR activity, reducing the differentiation of naive CD4^+^ cells to Th17 cells (28).

Besides, CNS2 contains the Treg cell-specific demethylation region (TSDR) of effector Tregs (41). The TSDR demethylation signature of Foxp3 serves as an effective predictor of dendritic protein function and immunosuppression. While CNS2 demethylation begins after the initiation of Foxp3 transcription, Foxp3 may be involved in this process. Indeed, CNS2-deficient Tregs achieve down-regulated Foxp3 expression. Thus, initial transcriptional activation of Foxp3 and subsequent CNS2 demethylation take on critical significance in establishing a faithful epigenetic memory of Foxp3 expression and ensuring Tregs lineage commitment (42). Furthermore, Foxp3 DNA methylation is reduced on Tregs, and Foxp3 reactivation leads to the up-regulation of CTLA-4, such that the Tregs function is normalized. CTLA-4 has been reported to be hypermethylated at the specific positions on Tregs of rheumatoid arthritis patients, such that the CTLA-4 expression is down-regulated (43).

### 2.3 Function of memory Tregs

During the inflammatory resolution phase of acute lung infection with influenza in a mouse model, Tregs, activated and expanded 50-fold, begin to shrink continuously and develop a pool of memory cells ultimately. The above-described memory Tregs rapidly expand 10-fold upon reinfection and secret considerable anti-inflammatory cytokine, IL-10 to suppress tissue damage and inflammation arising from recall expansion of memory CD4^+^ T cells (44).

Despite the relatively stable proportion of Tregs in the CD4^+^ T cell population throughout a person’s life, the proportion of memory Tregs varies. Nearly 80% of the Tregs in the umbilical cord blood of neonates are naive Tregs, probably because neonates only receive antigens from the mother through the placenta before birth. With age, the proportion of memory Tregs in the Tregs pool gradually increases due to stimulation by various external antigens, so CD45RO^+^ memory Tregs account for the vast majority in old age (45, 46). As indicated by existing research, almost all Tregs in adult skin express CD45RO, the proportion of Tregs expressing CD45RO in fetal skin is significantly down-regulated by comparison (47, 48).

When self-antigens persistently stimulate tissues, the first responders rushing to the scene are not effector T cells (Teffs) but Tregs (27). As an important subgroup Tregs, memory Tregs also have stronger local tropism and migration ability than naive Tregs (49). Memory Tregs can rapidly migrate to non-lymphoid tissues, such as lung and liver, to control local tissue immune damage (50).

Epigenetic regulation of memory Tregs is complex since naive Tregs originating in the thymus or the periphery acquire immune memory function after antigenic stimulation. In kidney transplant patients, Foxp3 demethylation shows a significant correlation with the proportion of memory Tregs, and circulating memory Tregs in organ transplant tolerant patients express higher levels of CD39 and GITR and higher levels of Foxp3 demethylation and stronger suppressive function (51). The TSDR demethylation of CD25^int^CD45RA^-^ memory Tregs is less stable than that of naive Tregs since their demethylation is reduced notably and accompanied by down-regulated Foxp3 expression 2 weeks after expansion. Thus, the immunosuppressive capacity of amplified CD25^int^CD45RA^-^ memory Tregs is reduced, whereas it is nearly consistent with that of naive Tregs (52).

### 2.4 Molecules related to memory Tregs function

Chemokine receptor type 7 (CCR7)^-^ memory cells are capable of migrating to inflamed tissues and displaying immediate effector functions. CCR7^+^ memory cells lack immediate effector function, whereas they can efficiently activate dendritic cells and differentiate into CCR7^−^ effector cells when re-stimulated (53). Accordingly, memory cells with CCR7^+^ and CCR7^−^ are defined as central and effector memory cells, respectively (54).

In accordance with CD31 expression, Tregs can fall into CD31^+^ recent thymic emigrants and CD31^-^ mature subgroups (55). Moreover, this subtyping can be applied to the classification and functional judgment of memory Tregs.

Human leukocyte antigen (HLA)-DR belongs to the HLA class II molecules, which is expressed on the surface of various immune cells and can be considered an activation marker of Tregs (56). HLA-DR-positive Tregs express a high level of Foxp3, such that many studies have divided memory Tregs into DR^+^ memory Tregs and DR^-^ memory Tregs subsets by HLA-DR.

GITR refers to a member of the tumor necrosis superfamily, can directly activate effector CD4^+^ and CD8^+^ T cells, thereby promoting antitumor immune responses (57, 58). Patients with slow progression of type 1 diabetes have the increased frequency of memory Tregs and considerable GITR expression in peripheral blood, whereas the immunosuppressive function of memory Tregs is significantly impaired (59).

PD-1 refers to an immunosuppressive molecule of the CD28 family ubiquitously expressed on immune cells (60). The dynamic balance of memory Tregs can be inhibited by PD-1, while anti-PD-1 treatment prevents the buildup of memory Tregs (61). Wang et al. suggested that RPL patients had a lower proportion of PD-1^+^ memory Tregs in their peripheral blood than healthy pregnant women, suggest fetal antigen stimulation can cause increased expression of PD-1 and affect the proliferation, accumulation, or function of memory Tregs (18).

CD80/CD86 are B7 ligands that can compete with CTLA-4 for binding to CD28 (60, 62). Dominik et al. suggested that the most active mTreg clusters had significantly increased expression of CD80 and CD86, and both CD80^+^ and CD86^+^ memory Tregs showed normal Foxp3 and Helios expression profiles. In addition, CD80 and CD86 were also observed to be co-expressed with Tregs activation markers CTLA-4 and HLA-DR (33).

## 3 Memory Tregs in normal pregnancy

Tregs take on critical significance during pregnancy as the maternal immune system requires immune tolerance to the semi-allogeneic fetus (63–65). Tregs circulate in maternal peripheral blood, and they are capable of converging in basal decidua and parietal decidua at the maternal-fetal interface during pregnancy (66, 67). Tregs involved in the regulation of autoimmunity and tolerance to the fetus by suppressing the maternal immune response (68). Tregs-mediated immune tolerance emerges in the pre-implantation phase of early pregnancy, and decidual Tregs continuously expand in the first and second trimesters, such that subsequent pregnancy maintenance is significantly facilitated till they decline before delivery (68–70). Memory Tregs are the main component of the reproductive system of healthy women (parous and nulliparous combined), with memory Tregs accounting for about 70% of the Tregs pool in peripheral blood and 97.9% in endometrium (71). Existing research on mice suggested that maternal Tregs targeting fetal-specific antigens are produced extrathymic and then recruited onto the maternal-fetal interface by CNS1 dependent manner with high priority (72, 73). Chen et al. suggested that Tregs were rapidly recruited to uterine draining lymph nodes during pregnancy and activated on the first day after embryo implantation through the mice model. They express the activated/memory Tregs (amTregs) subset markers CD44^high^CD62L^low^ and at least in part autoantigen-specific (74).

In addition, as depicted in **Figure 1**, naive Tregs in the peripheral blood of healthy pregnant women display a notable tendency to differentiate into memory Tregs, which takes on vital significance in the maintenance of pregnancy. Besides, the initiation of delivery is correlated with the significant breakdown of this differentiation tendency to memory Tregs (75). Miriam et al. suggested that during the first trimester of pregnancy, the proportions of thymus-immigrant regulatory T cells (CD31^+^ naive Tregs) and CD31^+^ memory Tregs were significantly decreased, while CD45RA^-^CD31^-^ memory Tregs were correspondingly enhanced (76). Abnormal Tregs proportion and function breaks the adaptive immunity to the fetus, leading to a wide variety of pregnancy complications (e.g., RPL, PE and gestational diabetes (GDM)) (77–79). There are dynamic changes among the subpopulations of the Tregs pool during pregnancy, especially in the 10-20th week of gestation, where the abnormal differentiation of naive Tregs to memory Tregs shows a correlation with the pathogenesis of PE (75, 80).

### 3.1 The activation of memory Tregs in pregnancy

The proportion of memory Tregs subgroups takes up the major position of Tregs pool in the peripheral blood or endometrium of reproductive women, and memory Tregs outperform naive Tregs in the immune regulation function, which undoubtedly takes on critical significance in the success of pregnancy (**Figure 1**) (46, 71). Compared with the powerful functions of memory Tregs, considerable mysteries should further be explored in their formation, activation, and maintenance during pregnancy.

The maternal immune system is stimulated by paternal antigens in semen during copulation while developing immune tolerance (7, 81, 82). After mating between sterile female mice and 2W1S^+^ male mice, the number of 2W1S^+^ Tregs is up-regulated at a lower level than normal pregnant mice, suggesting the formation of paternal antigen memory Tregs carried by semen (17).

In general, the expression of Foxp3, the stability of lineage, and the expression of numerous landmark genes in Tregs are dependent on TCR signaling (32). However, the stimulation of TCR signaling alone is insufficient to maintain the function of Tregs and exert an effective immune regulation function (83). IL-2 is capable of supporting Tregs with survival signals while increasing their immunosuppressive effects, and IL-2 has been confirmed as a vital factor for the activation of memory Tregs (84, 85). Memory Tregs express high levels of IL-2Ra and IL-7Ra. Unlike IL-2-dependent pTregs and tTregs in secondary lymphoid tissue, skin-resident memory Tregs are activated by IL-2, and then their maintenance is determined by IL-7 (85, 86). However, the dependence of memory Tregs on IL-7 has only been confirmed in the skin, and no conclusive evidence has been reported during pregnancy. In a mice model, blocking IL-10R significantly suppresses the ability of memory Tregs to inhibit memory CD4^+^ T cell recall expansion and the accompanying immunopathological variations *in vivo*, suggesting that IL-10 may play a certain role in and enhancing the immune regulation of memory Tregs (44).

Besides the above-mentioned factors that may affect the memory function of immune regulation, memory Tregs may show a correlation with the “pregnancy-induced microchimerism” existing in maternal (87). During pregnancy, the close bond between mother and child contributes to a bilateral exchange of small numbers of cells across the placenta (88). Fetal cells with semi-allogeneic gene enter into maternal blood circulation during pregnancy, and the above-mentioned cells persist long after delivery (89). As indicated by recent findings, the presence of the above-described microchimeric cells expressing fetal-specific antigenic features does not arise from coincidence, whereas it is deliberately retained in the maternal while ameliorating the outcome of re-pregnancy by promoting the adaptation to paternal genetic material (90).

During subsequent pregnancies, with the Tregs pool generally maintaining a homeostasis, the proportion of memory Tregs varies since the pregnancy progresses and functions differently at the respective stage. Impaired fetal tolerance in the first trimester may trigger spontaneous abortion, some minor fluctuations may result in preterm birth or fetal growth restriction, and interruption of fetal tolerance in the third trimester may cause the occurrence of PE/eclampsia or even stillbirth (91). Compared with non-pregnant women, the proportion of HLA-DR^+/-^ memory Tregs in peripheral blood is increased rapidly during the first 7 weeks of pregnancy. The above-mentioned phenomenon is most likely to rapidly enhance the maternal immune tolerance function to complete the vital procedure of embryo implantation. Subsequently, memory Tregs tend to be decreased, whereas they remain at a high level (46). As the fetus leaves the mother after parturition, fetal-specific antigens in maternal peripheral blood are decreased rapidly with the termination of maternal-fetal exchange, whereas they do not vanish.

### 3.2 Memory Tregs and second pregnancy

The re-pregnancy process after the first delivery may be most benefited from the immune regulation of memory Tregs, since their memory function contributes to a rapid response to the re-emergence of fetal-specific antigens. Moreover, the fetal resorption rate after partial FOXP3^+^ cell ablation in re-pregnant mice is notably lower than first pregnancy (17). Thus, in a prospective cohort study with 763,795 pregnant women recruited, the risk of PE during the first pregnancy is 4.1%, and the risk is reduced to 1.7% during the second pregnancy (92). As indicated by the above result, the first pregnancy process can significantly protect the second pregnancy, whereas this significant protection will not be constant. This protective effect can be efficient only when the paternal antigens of the two pregnancies remain unchanged. If the partner changes in second pregnancy, the efficiency of this immune regulation will decline significantly (93). Notably, if a woman has PE during the first pregnancy, the risk of PE during the second pregnancy is not reduced (94). Furthermore, the risk of preterm birth in re-pregnancy is significantly elevated if the first pregnancy exhibits pregnancy complications or adverse pregnancy outcomes (93). The susceptibility to pregnancy complications in re-pregnancy is increased with the course of the first pregnancy, and it is very likely that a complex mechanism exists behind them.

Maternal CD8^+^ T cells begin to expand systematically and initiate immune rejection under the effect of paternal antigen stimulation during the first pregnancy; such expansion continues even after parturition (95). During the second pregnancy of mice, maternal memory CD8^+^ T cells are not expanded when stimulated by fetal antigens again. The expression of PD-1 and LAG-3 on the surface of the above-mentioned cells is up-regulated, and their immune rejection function are exhausted (96). Besides, decidual NK cells (dNK) can exhibit special innate memory capabilities for first pregnancy, such that the above-described cells are termed “pregnancy-trained dNK cells”. Memory dNK cells achieve the high expression of NKG2C and ILT2 during re-pregnancy while producing considerable interferon–γ (IFN–γ) and vascular endothelial growth factor-α (VEGF-α) to more effectively support angiogenesis and endometrial vasculature remodeling during embryonic development. The above-mentioned measures can facilitate the success of subsequent pregnancies (97).

The effect of first pregnancy on the function of memory Tregs has been rarely investigated, and most studies have placed a focus on memory Tregs expansion rather than functional changes during the second pregnancy. Granne et al. suggested that Tregs in the endometrium of parous women expressed 38.8% of uniquely genes, while Tregs in the endometrium of nulliparous women expressed only 1.8% of uniquely genes. Since memory Tregs make up 97.9% of the endometrial Tregs pool in healthy women, the experience of a successful pregnancy may more significantly affect memory Tregs than we expect (71).

However, although existing evidence has confirmed the establishment of a memory immune protection system during pregnancy, the specific functions of memory Tregs after re-stimulation with fetal-specific antigens during subsequent pregnancy have been scarcely investigated. Accordingly, the exact mechanism by which the first adverse pregnancy triggers the risk of pregnancy complications in subsequent pregnancies remains unclear. However, a speculation is made in accordance with the existing research. The possible reason for the above result is the abnormal function of memory Tregs during the first adverse pregnancy. Consequently, a well-functioning fetal protection system cannot be developed, or immune killer cells (e.g., memory CD8^+^ T cells) have hyperfunction, or the mother is susceptible to placental dysfunction. In general, the truth of the relevant issues should be revealed by in-depth research.

## 4 Memory Tregs in pregnancy complications

### 4.1 Memory Tregs in PE

The occurrence of PE covers multiple factors (e.g., placental defects, vascular damage, and immune imbalance). To be specific, the immunological property of PE refers to the reduced function of the adaptive immune system (98).

As indicated by a meta-analysis conducted by Green et al., the number of Tregs in the maternal peripheral blood of patients with PE is lower compared with healthy pregnancies (99). The Tregs pool is subjected to the systemic and localized expansion during human pregnancy. To be specific, clonally expanded effector Tregs are increased in the decidual. During human pregnancy, the Tregs pool is subjected to a systemic and localized expansion, with an increased presence of clonally expanded effector Tregs in the decidua after fetal antigens are recognized. Existing research has suggested that the clonal effect of decidual effector Tregs is diminished in PE patients when compared with healthy third-trimester women (100).

With the reduced proportion of CD45RA^+^CD31^+^ Tregs during healthy pregnancy, the ratio of CD45RA^+^CD31^+^/CD45RA^+^CD31^-^ Tregs declines, such that the immunosuppressive function of the naive Tregs pool is increased. CD45RA^+^CD31^+^ Tregs tend to differentiate into CD45RA^−^CD31^−^ memory Tregs during healthy pregnancy. However, this differentiation tendency is disrupted in PE patients, and the proportion of memory Tregs in Tregs declines, such that the immune regulatory system is impaired (75, 76). Steinborn et al. examined HLA-DR as an activation marker of memory Tregs. As indicated by their results, compared with healthy pregnant women, the proportion of DR^+^CD45RA^-^ memory Tregs in the peripheral blood of PE patients is increased, whereas the immunosuppression of total Tregs is significantly reduced (80).

Kieffer et al. suggested that memory Tregs in decidua parietalis of early-onset PE (PE onset before 34 weeks) patients are significantly increased compared with normal pregnant women, and the effector memory Tregs is mainly up-regulated. In contrast, memory Tregs in decidua parietalis of patients with late-onset PE (PE onset after 34 weeks) vary less, whereas central memory Tregs are significantly reduced. The proportion of effect memory Tregs is also up-regulated in decidua parietalis of late-onset PE, whereas it is not statistically significant. The decidua parietalis of early-onset PE patients express higher levels of IFN-γ and IL-2 mRNA than normal pregnant women. Overall changes in memory Tregs are smaller in decidua basalis in both early-onset PE and late-onset PE patients (101).

### 4.2 Memory Tregs in GDM

GDM shows a correlation with numerous maternal and fetal adverse pregnancy outcomes, including caesarean section, PE, macrosomia, intrauterine growth retardation, delayed neonatal brain maturation, and neurobehavioral abnormalities (102, 103). Among those with a previous diagnosis of GDM, 22.6% would develop diabetes within the next 8 years (104). Besides the well-known glucose intolerance and insulin resistance, GDM is also characterized by chronic systemic inflammation and enhanced humoral immune responses (105, 106). The above-described characteristics are also present in non-pregnant diabetic patients. The peripheral blood of both type 1 diabetic mice and patients was flooded memory Tregs with high expandation but impaired function, which showed increased GITR expression, decreased CD39 expression and suppressed clonotype expansion of TCR (59, 107, 108).

Schober et al. suggested that although the total Tregs pool in the peripheral blood of GDM patients was not decreased, but the immunosuppressive activity of Tregs was significantly reduced compared with healthy pregnant women. The proportion of naive Tregs decreased and the proportion of memory Tregs increased in both diet-adjusted and insulin-dependent GDM patients. The discrimination was that the proportion of HLA-DR^-^ memory Tregs was significantly increased in patients with diet-adjusted GDM, whereas the proportion of HLA-DR^low+^ and HLA-DR^high+^ memory Tregs was significantly increased in patients with insulin-dependent GDM (109).

### 4.3 Memory Tregs in RPL

Parental chromosomal abnormalities, maternal thyroid disease or diabetes, endometrial changes, immune factors, and so forth may result in RPL (110). Wang et al. suggested that no significant difference exists in the proportion of memory Tregs in peripheral blood between healthy women and RPL patients at the non-pregnant state. After pregnancy, the proportion of memory Tregs in the peripheral blood of the two groups of women is significantly elevated, whereas the proportion of memory Tregs in RPL patients remains to be significantly lower than healthy women (18).

The differential expression of immune checkpoint molecules on memory Tregs is likely to indicate variations in their function and expansion capacity. Granne et al. using RNA-sequencing techniques reported significant differences in the transcriptional profiles of Tregs of the endometrium whether comparing primary RPL to nulliparous women or secondary RPL to parous women. To be specific, the expression level of TIGIT on the surface of Tregs in RPL patients is notably lower than that in normal women, whereas the levels of Foxp3, Helios and CTLA-4 are not significantly different (71). After pregnancy, whether in RPL patients or healthy women, PD-1, CCR6, and HLA-G expression on memory Tregs in peripheral blood is significantly up-regulated compared with that in non-pregnancy, and the expression levels of RPL patients after pregnancy are still lower than those in normal pregnancy female (18).

### 4.4 Memory Tregs in preterm birth

There are various factors leading to preterm birth (e.g., PE, fetal growth restriction, infection, and short cervix that can all serve as predictors of preterm birth) (111). In mice, depletion of Tregs during the late gestation of pregnancy lead to premature delivery and adverse neonatal outcomes, in the process, the effect of Tregs exhaustion during the second pregnancy on premature delivery was smaller than that of the first (14). The mechanism of Tregs depletion leading to adverse perinatal outcomes comprises tissue-specific immune response, mild systemic maternal inflammation, and disorders of placental development. For premature neonates, the percentage of Tregs in cord blood is negatively correlated with gestational age, whereas the expression level of CTLA-4 on the surface of memory Tregs is up-regulated with gestational age (112). Steinborn et al. suggested that DR^-^CD45RA^+^Tregs were increased in the peripheral blood of premature women, while DR^-^CD45RA^-^Tregs and DR^low+^CD45RA^-^Tregs were decreased (80).

### 4.5 Memory Tregs and assisted reproductive technology

As infertility arising from various reasons affects nearly 15.5% of couples of childbearing age, assisted reproductive technology turns out to be the first choice for treating infertility (113). Schlossberger et al. analyzed the correlation between the number and function of memory Tregs and *in vitro* fertilization (IVF)/intracytoplasmic sperm injection (ICSI) success through a prospective study. The proportion of naive Tregs in peripheral blood of pregnant patients after IVF/ICSI treatment is elevated, the proportion of DR^-^ memory Tregs is reduced, and the proportion of DR^+^ memory Tregs is also down-regulated, whereas it is not statistically significant. It is noteworthy that naive Tregs of pregnant patients exhibit the significantly greater immunosuppressive ability than non-pregnant patients, whereas the immunosuppressive ability of DR^+^ memory Tregs is significantly lower than that of non-pregnant patients. The increase of DR^+^ memory Tregs begins to retard gradually from the age of 40, suggesting the powerless of naive Tregs to transform into DR^+^ memory Tregs. In this study, the age of non-pregnant patients after IVF/ICSI is notably higher than pregnant patients, suggesting that the reduced immunosuppressive activity of DR^+^ memory Tregs in non-pregnant patients may be affected by age (46).

**Figure 2:**
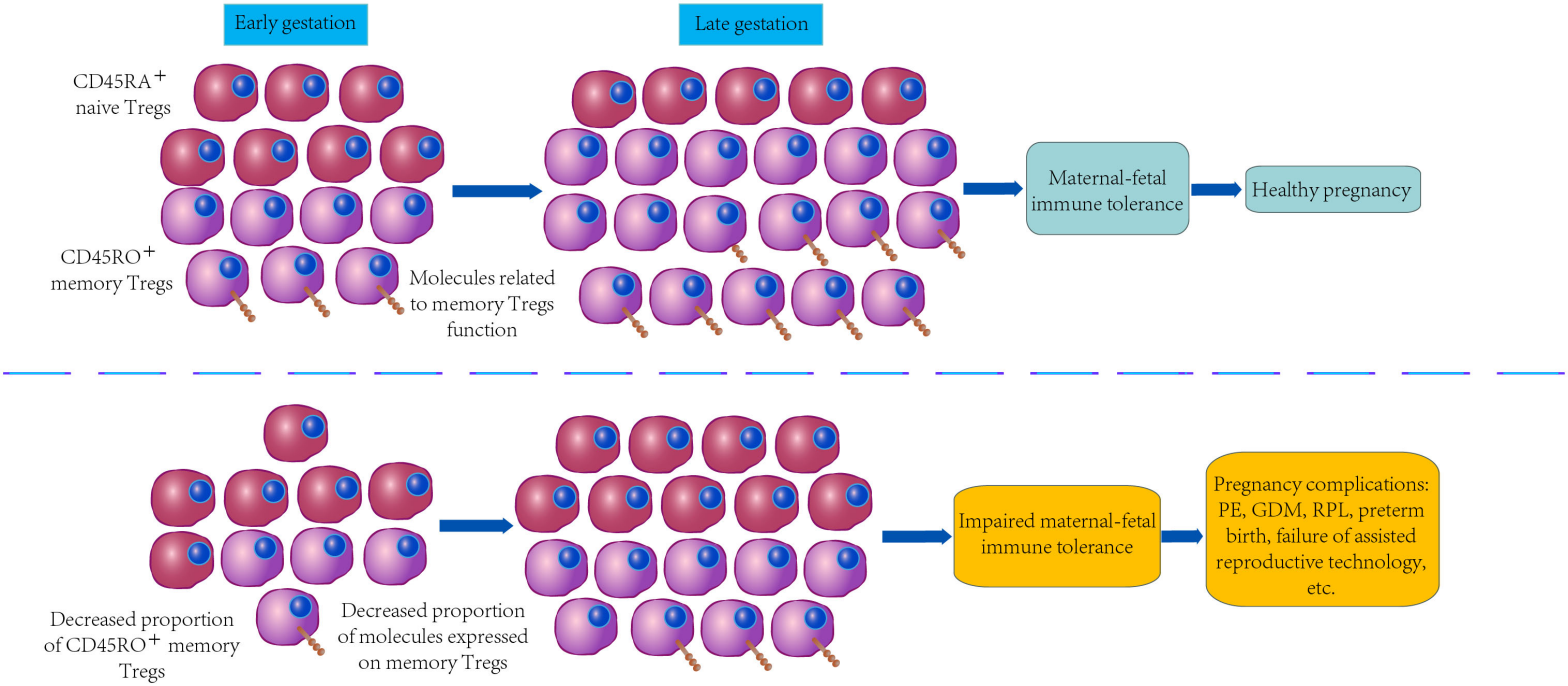
Differences of memory Tregs in normal pregnancy and pregnancy complications.

As depicted in **Figure 2**, the down-regulated expression of the above-mentioned vital checkpoint molecules on memory Tregs may play a certain role in the pathogenesis of PE, GDM, RPL, preterm birth and failure of assisted reproductive technology by reducing stability, proliferation, amplification, immunosuppression, or chemotaxis.

### 4.6 Memory Tregs and reproductive potential

Under multiple factors, the first pregnancy age of women has displayed an increasing trend in the last decades (114). Although ovarian function decline with the increasing age in women of reproductive age, many young females are subjected to an irreversible process of premature ovarian failure or ovarian function decline, which is correlated with iatrogenic factors, genetic, environmental or immunological factors (115, 116). Rocamora-Reverte et al. suggested that the senility process was characterized by an altered composition of immune cells, and the structure of the total Tregs pool subsets was also altered (117). The features of senility comprise the increase of decrepit cells and the accumulation of inflammatory markers in peripheral blood (118). One of the significant characteristics of senility T cells were negative express for costimulatory molecule CD28 (119). The immunosuppressive function of CD28^-^ Tregs is significantly reduced, and they play a certain role in the pathogenesis of a wide variety of immune-associated diseases (e.g., rheumatoid arthritis and multiple myeloma) (120, 121). On the other hand, the proportion of DR^+^ memory Tregs in the peripheral blood of women of childbearing age shows a positive correlation with age, and the velocity of growth rate tends to be decelerated after the age of 40 (46).

Kahindo et al. suggested that the level of DR^+^ memory Tregs in the peripheral blood of women aged 40-49 years is significantly higher than that of women aged between 20 and 39 years. The level of DR^+^ memory Tregs shows a significant positive correlation with the level of blood follicle-stimulating hormone (FSH) and a significant negative correlation with the level of anti- müllerian hormone (AMH) and antral follicle count (AFC). Furthermore, the level of CD28^-^ Tregs is negatively correlated with the level of AFC (122). Thus, as indicated by the above-described results, Tregs subpopulations are associated with ovarian reserve markers, and the composition of Tregs pool subpopulations is expected to be useful in assessing ovarian function and predicting subsequent reproductive potential.

## 5 Immunotherapeutic modalities for memory Tregs

Memory Tregs play a key role in suppressing excessive immune responses and maintaining immune homeostasis, and defects in their function lead to imbalance of the immune system and a variety of immune-associated diseases. Accordingly, many researches actively explored therapeutic modalities to increase the number of memory Tregs and activate their immunosuppressive capacity, with the expectation that the above-mentioned measures could become effective treatment modalities for immune-associated diseases.

### 5.1 Low-dose of IL-2

Tregs express high levels of IL-2Ra (CD25), and IL-2 can facilitate the proliferation, maturation, and anti-apoptotic effects of Tregs to enhance the immune regulatory function (123). Asano et al. conducted murine research. As indicated by the result, s low-dose IL-2 selectively increases the proportion of Tregs while promoting PD-1 expression and maintaining the stable expression of other suppressive molecules (e.g., CTLA-4, LAG-3, and TIM-3), which are particularly expressed on CD44^+^ CD62L^+^ central memory Tregs (124).

Mhanna et al. performed TCR-sequencing on CD4^+^ T cell subsets of the spleens from normal C57BL/6 (B6) and non-obese diabetic (NOD) mice (107). The TCR repertoire of the CD4^+^ Foxp3^+^ CD44^high^ CD62L^low^ amTregs are the least diversity. In contrast, NOD mice exhibit a significantly higher repertoire diversity of amTregs TCR compared with that of B6 mice. Under this property of NOD mice, amTregs are difficult to activate, and minor immunomodulatory effects are exerted. When stimulated by sustained low-dose IL-2, NOD mice exhibit a significantly higher proportion of amTregs, while the clonotypic expansion of amTregs is rejuvenated.

Cunningham et al. injected high, medium and low doses of IL-2 to PE rat models. As indicated by their results, PE rats injected with low-dose of IL-2 have decreased blood pressure and the down-regulated levels of v Chimeric antigen receptors asoconstrictor peptide endothelin-1, while the fetus is adversely affected (125). Thus, this treatment modality may exert a therapeutic effect on pregnancy complications by enhancing the functional activity of memory Tregs, whereas the specific evidence should still be verified in the future.

### 5.2 mTOR inhibitors

As mentioned before, sustained glycolytic activity inhibits memory formation, whereas inhibition of glycolysis promotes memory cells development (126). Rapamycin, i.e., a selective inhibitor of mTOR, is capable of increasing lipid oxidation and reducing glycolysis, and then suppressing the immune response of Teffs and facilitating the generation of Tregs (36, 127, 128). Given the metabolic similarity between memory cells and Tregs, one theory holds that if naive cells stimulated by high level mTOR signal would differentiate into effector Tregs, while stimulated by low level mTOR signal would differentiate into memory Tregs (129). Short term inhibition of mTOR alleviates the negative regulation of Tregs by costimulation domain 4-1BB tonic signaling (130).

Up-regulated PI3K/Akt/mTOR signal pathway can suppress Foxp3 expression, induced aberrant immunosuppressive function of Tregs and ultimately caused spontaneous miscarriage or fetal malformations in mice (131). Royster et al. demonstrated that Tregs exhausted mice exhibited a significant increase in litter size after rapamycin injection treatment (132). But the long-term use of rapamycin have risks such as cancer, stroke, cerebral infarction, blindness and premature death (133). Zhang et al. suggested that the combined administration of low-dose rapamycin and higher dose IL-2 can reduce side effects while enhancing Tregs expansion and immunoregulatory function (134).

### 5.3 Other potential treatment methods

Adoptive transfer of Tregs is a promising high-effectiveness strategy for treating diseases mediated by the impaired function of Tregs. Such therapeutic modalities comprise isolating differentiated Tregs *in vivo*, expanding Tregs *in vitro*, or generating inducible Tregs *in vitro* and transfer into body subsequently (135). Wang et al. built CBA/J×BALB/c normal pregnancy mice model and triggered an increase in the miscarriage rate through the transvaginal injection of rIL-17. After the adoptive transfer of purified Tregs from healthy pregnant mice, the increased miscarriage rate in the treated mice model is reversed, and the levels of TGF-β and IL-10 in the decidual Tregs are up-regulated (136). Thus, Tregs adoptive transfer therapy may be promising in treating miscarriage due to inflammation. However, this treatment method has only been proven to be effective in inbred animals of the identical strain, and whether it can be employed in the clinical treatment of humans should be investigated in depth. Furthermore, memory Tregs are a subpopulation of Tregs with a low number, and the sorting and purification of memory Tregs may be a laborious task for this reason.

Chimeric antigen receptors (CARs) are a series of engineered fusion proteins, which comprise extracellular single-chain variable fragment recognizing antigens, intracellular immunoreceptor tyrosine-based activation motifs, transmembrane domain and costimulatory domain. They are capable of redirecting the specificity and function of T lymphocytes and other immune cells (137, 138). Existing research has reported that CAR-Tregs achieve positive outcomes in treating immune-associated diseases (e.g., graft-versus-host disease, type 1 diabetes, inflammatory bowel disease and other immunity-associated diseases) (139–141). CAR-Tregs show numerous advantages (e.g., being capable of maintaining a stable phenotype and function, being less dependent on IL-2, preferentially migrating to targeted tissues, and exerting stronger and more specific immunosuppressive effects over polyclonal Tregs) (142). However, with the aim of constructing highly specific and effective memory CAR-Tregs, antigens the CARs targeted should be selected, and specific antibodies should be developed. Thus, this construction process is undoubtedly a tough challenge for several diseases (142).

## 6 Conclusion

In brief, Tregs are activated after being stimulated by cytokines (e.g., fetal-specific antigens in paternal semen, embryo cells, and pregnancy-induced microchimerism) and differentiate into embryonic antigen-specific memory Tregs. The differentiation of memory Tregs is regulated by numerous factors (e.g., TCR signal stimulation, transcription factor interaction, mTOR signal, and methylation). It is more certain that the reduced number and abnormal function of maternal memory Tregs show a correlation with a wide variety of pregnancy complications and adverse outcomes, whereas rare effective treatment methods have been proposed. Accordingly, the characteristics of memory Tregs in normal pregnancy and pathological pregnancy should be further researched, and the exploration of molecule markers correlated with memory Tregs function can be conducive to revealing the pathogenesis of pregnancy-associated diseases and providing a strategy for gaining insights into subsequent clinical diagnosis and treatment.

## Author contributions

ZC completed the data collection and writing of the manuscript. YZ polished the language of the manuscript. JK-K completed the manuscript proofreading and content guidance. WW provided the manuscript ideas and completed the manuscript proofreading. All authors contributed to the article and approved the submitted version.
